# Combination of SPH and SP80 prolongs the lifespan of *Bacillus subtilis* natto to enhance industrial menaquinone-7 biosynthesis

**DOI:** 10.3389/fmicb.2025.1578160

**Published:** 2025-06-04

**Authors:** Liu-xiu Hu, Yu Chen, Hong-ping Zhou, Wei Tao, Xu-li Gao, Chuan-chao Wu, Hui-min Zhang, Ru-meng Han, Yu-qi Li, Yan Liu

**Affiliations:** ^1^College of Biology and Food Engineering, Anhui Polytechnic University, Wuhu, China; ^2^Anhui Zhang Hengchun Pharmaceutical Co., Ltd, Wuhu, China; ^3^Wuhu Green Food Industry Research Institute Co., Ltd, Wuhu, China; ^4^College of Chemistry and Environmental Engineering, Anhui Polytechnic University, Wuhu, China; ^5^Anhui Engineering Laboratory for Industrial Microbiology Molecular Breeding, Wuhu, China

**Keywords:** menaquinone-7, lifespan, soy protein hydrolysate, span 80, *Bacillus subtilis* natto

## Abstract

Menaquinone-7 (MK-7) production from renewable feedstocks using *Bacillus subtilis* natto provides a promising pathway toward sustainability. However, MK-7 yields are often limited by poor microbial cell viability. In this study, a novel fermentation strategy aimed at prolonging the lifespan of *B. subtilis* natto was investigated to enhance MK-7 biosynthesis. The results showed that the combination of soy protein hydrolysate (SPH) and Span 80 (SP80) increased intracellular and extracellular MK-7 yields by 5.6- and 7.2-fold, respectively, compared to the SP-based medium. This enhancement was associated with an extended lifespan of *B. subtilis* natto, as evidenced by increased optical density and cell length, and a reduced cell death rate—13.5% in the stationary phase compared to 39.5% in the SP-based medium. Moreover, a significant correlation was observed between key MK-7 biosynthetic genes and lifespan-related genes. The expression of growth-related autolysis genes s*kfF* and *sdp,* which contribute to reduced cannibalism, was downregulated by 4.89- and 5.19-fold, respectively. In contrast, expression of the cell division genes *ftsZ* and *ftsL*, which promote cell division, was upregulated by 7.52- and 6.31-fold, respectively. Improved fermentation performance was attributed to a 2.26-fold increase in oligopeptides (Mw < 1 kDa) and moderate levels of amino acids, particularly Phe, Arg, and Glu, derived from the enzymatic hydrolysis of soy protein by Protamex 1.6, present in the SPH+SP80 medium. Meanwhile, the prolonged lifespan also promoted MK-7 biosynthesis by upregulating key membrane-associated genes such as *menA* and *menD*, which showed 1.43- and 1.47-fold increases in expression, respectively. This contributed to improved MK-7 precursor availability and enhanced MK-7 assembly efficiency in *B. subtilis* natto. Collectively, these results indicate that prolonging the lifespan of *B. subtilis* natto using SPH+SP80 represents a promising and effective strategy for improving industrial MK-7 production.

## 1 Introduction

Menaquinone-7 (MK-7), a highly valuable lip-soluble form of vitamin K_2_, features a ring structure comprising a 2-methyl-1,4-naphthoquinone core and a side chain of seven isoprene units. MK-7 has been identified as a component of microbial plasma membranes, where it plays an important role in electron transport and oxidative phosphorylation (Wu et al., 2021). In the current era of heightened health awareness, MK-7 has gained increasing attention for its efficacy in preventing and treating various diseases, especially those affecting bone and blood health (Liu et al., 2021). Interestingly, recent studies have shown that MK-7 acts as a potent anti-ferroptotic compound (Mishima et al., 2022), which is associated with the prevention of numerous age-related diseases, including osteoporosis, cardiovascular disease, cancer, Alzheimer’s disease, and diabetes. With the global rise in aging populations, the demand for MK-7 has grown substantially due to its broad therapeutic potential.

Several bacteria, including *Escherichia coli*, *Lactobacillus*, *B. subtilis* natto, and *Bacillus amyloliquefaciens,* have been employed to produce MK-7. *B. subtilis* natto became the dominant strain due to its safety, short growth cycle, and relatively higher MK-7 yield (Table 1). These bacteria are commercially available worldwide as nutritional supplements in food industrial production. However, the high production cost caused by the low yield has been the greatest challenge in the biosynthesis of MK-7.

**Table 1:** Different strategies used for the production of MK-7.

Strain	Strategies	Original state of the strain	Type	Fermentation time	Titer	Productivity	References
*Bacillus subtilis* natto OUV23481	UV and analog resistance (HNA, pFP, mFP, β-TA)	Isolated from a commercial natto supplied by Asahi Freshia Co., Ltd.	MK-7	16 h	3,438 μg/100 g	2.149 μg/g·h^−1^	Tsukamoto et al. (2001)
*B. subtilis* (natto)-P15-11-1	Strain mutation (NTG, HNA, and N^+^ ion-beam) media optimization	The strain *B. subtilis* (natto)-2–6 was isolated from the laboratory	MK-7	70 h	3.593 mg/L	0.051 mg/L·h^−1^	Song et al. (2014)
*B. subtilis*	Strain mutation (1-naphthol and Tween80)	*Bacillus subtilis* MTCC 2756 was procured from a Microbial Type Culture Collection	MK-7	24 h	14.4 μg/mL	0.600 mg/L·h^−1^	Puri et al. (2015)
*Bacillus licheniformis*	Strain mutation (kanamycin and shikimate)	Received from H. Gest	MK-7	10 h	0. 3 nmol/mL	0.019 mg/L·h^−1^	Goodman et al. (1976)
*B. subtilis* 168	Overexpression of *dxs, dxr, idi,* and *menA*	The MK-7-producing strains were isolated from commercially available natto	MK-7	6 days	50 mg/L	0.347 mg/L·h^−1^	Ma et al. (2019)
*B. subtilis* 168	Overexpression of *menA, dxs, dxr, yacM, yavN,* and *glpD*, and deletion of *dhbB*	Strains were derived from the laboratory constructed strain *B. subtilis* MK3-MEP123-Δ*dhbB*	MK-7	120 h	69.5 mg/L	0.579 mg/L·h^−1^	Yang et al. (2019)
*Bacillus amyloliquefaciens*	Overexpression of *menA*, *menC*, *menD*, *menE*, *menH* and *hepS*	*B. amyloliquefaciens* Y-2 and *B. amyloliquefaciens* W-21 were isolated from Chinese fermented beans	MK-7	24 h	273 mg/g DCW	11.375 mg/g·h^−1^	Xu et al. (2017)
*E. coli*	Overexpression of *idi*, *menA*, and *ubiE* and fine-tuning the expression of HepPPS, MenA, and UbiE	rrnBT14Δ*lacZWJ*16*hsdR*514Δ*araBAD*AH33Δ*rhaBA*	MK-7	52 h	13.6 μM	0.169 mg/L·h^−1^	Gao et al. (2021)
*E. coli* DH5α	Overexpression of *fatB* from *Umbellularia californica*	*E. coli* strain FM3-1709 was obtained as a 1-hydroxy- 2-naphthoate-resistant mutant and further mutated by nitrogen ion beam irradiation	MK	120 h	15.07 mg/L	0.125 mg/L·h^−1^	Liu et al. (2017a), Liu et al. (2017b)
*Lactococcus lactis* ssp. *Cremoris* MG1363	Overexpression of *menA*, *mvk* and *preA*	*Lactococcus lactis ssp. cremoris* MG1363 was used as the host for expression studies	MK-7, MK-8 and MK-9	Overnight	680 nmol/L	0.036 mg/L·h^−1^	Bøe and Holo (2020)
*B. subtilis* natto strain CICC No. 25137	SPH and SP80 were added to the medium	Strains were screened by nitrosoguanidine and low-energy ion beam implantation	MK-7	84 h	52.9 mg/L	0.629 mg/L·h^−1^	This study

Lifespan engineering, which regulates the growth performance of cell factories by extending cellular longevity and productivity, represents a promising strategy to increase metabolic production. The lifespan of microorganisms is divided into the chronological (defined as the length of time that cells in stationary phase culture remain viable) and replicative lifespan (defined by the number of daughter cells produced before the decline phase) (Lindner et al., 2008). Inhibition of cell autolysis was a viable approach to extend the chronological lifespan.

For example, Zhao et al. combined knockout of peptidoglycan hydrolase-related genes, including *sigD*, *lytE*, *lytF*, *lytC*, *lytD*, and *lytG*, which significantly increased cell growth rate and alpha-amylase production (Zhao et al., 2018). Regulation of the replicative lifespan in *B. subtilis* not only promoted rapid cell growth and altered cellular morphology but also modulated cell division dynamics. The rate of mass transfer depended on the specific surface area of the cell, while the cell morphology could be partially regulated by the rate of cell division (Jiang et al., 2015). For example, a previous study inhibited cell filopodia by overexpressing the genes *ftsZ* and *ftsA* associated with the D phase of cytokinesis, resulting in 57.1% higher growth density, 30% higher specific growth rate, and 227% higher production of human leptin protein in *E. coli* (Jeong and Lee, 2003). Although there had been many successful cases of modifying the chronological lifespan (CLS) and replicative lifespan (RLS) of microorganisms to increase metabolite content, it was particularly important to find a convenient and fast way to regulate cell lifespan due to the unavailability of gene editing tools for *B. subtilis* natto.

As a cost-effective and easily accessible nitrogen source, plant protein hydrolysates have been applied to markedly enhance the activity and improve the lifespan of bacterial cells. The mechanisms may be that the addition of small molecular weight proteins, polypeptides, or amino acids in the growth medium changed membrane lipid composition, cell membrane fluidity, membrane permeability, and intracellular homeostasis, and thus impacted cell growth (Jin et al., 2022). In addition, surfactant was applied to increase microbial metabolite production, remove target products inside the cells, and avoid production inhibition (Liu et al., 2014). To the best of our knowledge, no detailed investigation has been conducted on the impacts of SPH and surfactants in improving bacterial lifespan and enhancing MK-7 synthesis. The inter-relationships between SPH and surfactant prolonging the cell lifespan and the relationship between lifespan prolongation and MK-7 biosynthesis were not elucidated thoroughly. These results will provide a novel perspective on the prolongation of lifespan in *B. subtilis* natto and better elucidate the mechanism of SPH and surfactant supplementation in MK-7 synthesis from multiple dimensions.

## 2 Materials and methods

### 2.1 The preparation of soy protein hydrolysates

Soy peptone (SP), soy protein, and Protamex1.6 were purchased from Jinan Weiduofeng Biotechnology Co., Ltd. (Jinan, China), Jiangsu Ruiduo Bioengineering Co., Ltd. (Jiangsu, China), and Keppel Biotechnology Co., Ltd. (Qingdao, China), respectively. The preparation of soy protein hydrolysate (SPH) is referred to as the previous method (Yi et al., 2024). The soy protein and distilled water with a mass ratio of 1:15 were pretreated at 80°C for 10 min, and then Protamex 1.6 (1.5%, w/w) was added for 24 h of hydrolysis at pH 7.0, 50°C. The hydrolysates were heated to 100°C for 10 min to inactivate the enzyme and then centrifuged at 8,000 *g* and 4°C for 10 min. The supernatants were designated as SPH.

### 2.2 Strains and culture conditions

*Bacillus subtilis* natto strain CICC No. 25137 was screened by nitrosoguanidine and low-energy ion beam implantation (Song et al., 2014) and stored in the China Center of Industrial Culture Collection. The bacterial cells were first cultivated in seed medium (3% soy peptone, 0.1% yeast extract, 5% glycerol, and 0.9% NaCl) for 15 h at 37°C with 150 rpm. Then, the bacterial cells were harvested and inoculated into the fermentation medium at a 3% inoculum size. The base medium consisted of 5% glycerol, 1% yeast extract, 0.4% K_2_HPO_4_, and 0.2% KH_2_PO_4_. To form different experimental groups, additional components were included as follows: 3% SP (SP group), 0.3% Span 80 (SP80 group), 3% SPH (SPH group), and a combination of 3% SPH and 0.3% SP80 (SPH+SP80 group). Fermentation was conducted at 37°C for 84 h, with the medium occupying 30% of the total volume of the fermentation flask.

### 2.3 Degree of hydrolysis, molecular weight (mw) distribution, and free amino acids

The SP, SPH, SP80, or SPH+SP80 and distilled water with a mass ratio of 1:15 were pretreated at 80°C for 10 min, and then protamex1.6 (1.5%, w/w) was added for 24 h of hydrolysis at pH 7.0, 50°C. The hydrolysates were heated to 100°C for 10 min to inactivate the enzyme and then centrifuged at 8,000 *g* and 4°C for 10 min. The supernatant was obtained and tested for degree of hydrolysis, Mw distribution, and free amino acids.

The o-phthalaldehyde method (Nielsen et al., 2001) was used to determine the degree of hydrolysis for SP and SPH. Serine was used for the standard curve. The degree of hydrolysis was measured using the following formula:


(1) $$W_{\text{serine}}=V\times C_{\text{serine}}\times N/(P\times X)$$

(2) $$\text{Degree of hydrolysis}\;(\%)=(W_{\text{serine}}-\beta )/(W_{\text{serine}}\times \alpha )$$

where C_serine_, W_serine_, X, V, P, N, α, and β were the concentration of serine per gram of protein (mmol/g), the total number of peptide bonds (mmol/g), the sample mass (g), the volume of hydrolysate (L), the protein mass fraction of the sample (%), the dilution ratio, and the correction factors of 0.97 and 0.34, respectively.

The high M_w_ distribution (>10 kDa) of the sample was analyzed by SDS-PAGE. Separation gels (1.5 mm thick, 81 × 74 mm) consisted of a 12% polyacrylamide resolving gel and a 4% polyacrylamide stacking gel. 20 μL of each sample, protein marker ZD101 (14.4 kDa-116.0 kDa), and 5 μL of loading buffer were loaded onto the gel and run at 150 V in a Bio-Rad Mini-Protean Tetra system. Finally, the gel was stained using Coomassie brilliant blue fast-stain solution and photographed.

The low Mw distribution (<10 kDa) of the sample was analyzed by high-performance liquid chromatography (Agilent, USA) equipped with a UV detector and a TSK gel G2000SWXL (7.8 × 300 mm, 250 mm). The mobile phase was water: acetonitrile (4:1, v/v) and flowed at 0.5 mL/min. The wavelength of 220 nm was used for detection and analysis.

The free amino acids were measured by an L-8900 amino acid analyzer (Hitachi, Tokyo, Japan). The sample pretreatment was performed using the previous method (Li et al., 2022).

### 2.4 Determination of biomass, MK-7 yield, cell viability, and membrane potential

The cell biomass of fermentation broth was measured by determining the optical density at 600 nm. The MK-7 detection method was the same as in our previous research (Zhou et al., 2023).

The cell viability was assessed by flow cytometry (Guava EasyCyte, America). The bacterial solution cultured in 84 h of fermentation was centrifuged at 4°C and 8,000 *g* for 3 min, washed 3 times with 0.2 M PBS buffer, and resuspended in buffer (the bacterial density was 10^7^–10^8^ CFU/mL). 100 μL of solution from each tube was transferred to new 1.5 mL microcentrifuge tubes, vortexed, and stained with Propidium Iodide (PI).

The membrane potential was measured by flow cytometry (Novo et al., 2000). Firstly, *B. subtilis* natto was incubated in SP, SPH, SP80, and SPH+SP80 fermentation medium at 37°C for 84 h. Then, the cells were harvested by centrifugation (5,723 *g*, 10 min, 4°C). The competent cells were prepared by resuspending in 1 mL of ice-cold buffer solution [distilled deionized water (DDW), 0.9% NaCl, or 1 mM MgCl_2_, 30 mM DiOC_2_(3)]. The mixtures were incubated in the dark at room temperature for 4 min after being mixed vigorously by vortex. Finally, the cells were centrifuged (8,000 *g*, 10 min, 4°C), washed three times with ice-cold DDW, and analyzed by flow cytometry.

### 2.5 Cell morphology observation

The cells were centrifuged after 48 h of fermentation and washed twice with 0.1 M phosphate buffer. The dried cells were then fixed with 2.5% glutaraldehyde at 4°C for 6–12 h. The cells were then washed three times with 0.1 M phosphate buffer (20 min each time) and dehydrated by gradient ethanol (30, 50, 70, 80, 95, and 100% ethanol once, 20 min each time). Finally, the cells were coated with gold powder and observed using a scanning electron microscope (Thermo Fisher Scientific Corp., USA).

### 2.6 Detection of surface tension, NADH and NAD^+^, and maintenance energy coefficient (MEC)

The Wilhelmy plate method was applied to test the surface tension of a solution containing SP, SPH, SP80, and SPH+SP80 (Ma et al., 2023). A liquid film formed because of surface tension when the specific plate with known length and height was fixed on the tensiometer and contacted with the gas–liquid interface. The surface tension was measured by both the liquid film’s gravitational force and the contact line’s length. The results for SP, SPH, SP80, and SPH+SP80 solutions were calibrated using ethanol and pure water. For each example, three measurements were performed at 25°C, and the average was obtained.

NADH/NAD^+^ levels of *B. subtilis* natto were measured referring to the previous method (Wang et al., 2019). NAD^+^ total (NAD^+^ and NADH) or NADH levels were quantified by a colorimetric assay at 450 nm using a SpectraMax i3x (Molecular Devices, USA). The NADH/NAD^+^ ratio was calculated using the following formula:


(3) $$\text{NADH}/{\mathrm{NAD}}^{+}\text{ratio}=C_{\text{NADH}}/(C_{\text{total}}-C_{\text{NADH}})$$

The MEC of *B. subtilis* natto was measured using the previous method (Ren et al., 2024). The strains were incubated continuously in a 500 mL fermenter containing a 50 mL fermentation medium. The culture was switched from batch to chemostat mode when the glycerol was almost consumed. The agitation rate, pH, and temperature were controlled at 400 rpm, 6.6, and 37°C. Glycerol consumption was continuously monitored until the growth of cells reached a steady state. The value of the MEC was expressed as the rate of glycerol consumption (m_gly_).

### 2.7 RNA isolation, library construction, sequencing, and data analyses

The samples of *B. subtilis* natto were obtained by centrifuging the fermentation broth after 6 days of fermentation. RNA was extracted by the RNAlock reagent, and total RNA was collected by the TRIzol reagent (Invitrogen, USA). The extracted RNA was detected with an Agilent Bioanalyzer 2,100 (Agilent Technologies, Palo Alto, CA, USA). The RNA library construction from samples was completed by the Shanghai Human Genome Research Center (Wu et al., 2021).

After obtaining the data, the SeqPrep/Sickle software first selected the clean data by removing the adapter reads and low-quality and short fragments from the original data. Second, the differentially expressed genes (DEGs) between SP and SPH+SP80 were identified by mapping the clean data to reference sequences. Finally, the DEGs were mapped to terms in the gene ontology (GO) and KEGG databases for analyzing the functions and pathways, which were judged through the log_2_ ratio ≥ 1 and FDR of ≤ 0.05.

## 3 Results and discussion

### 3.1 Effect of MK-7 on microbial cell viability

**Figure 1:**
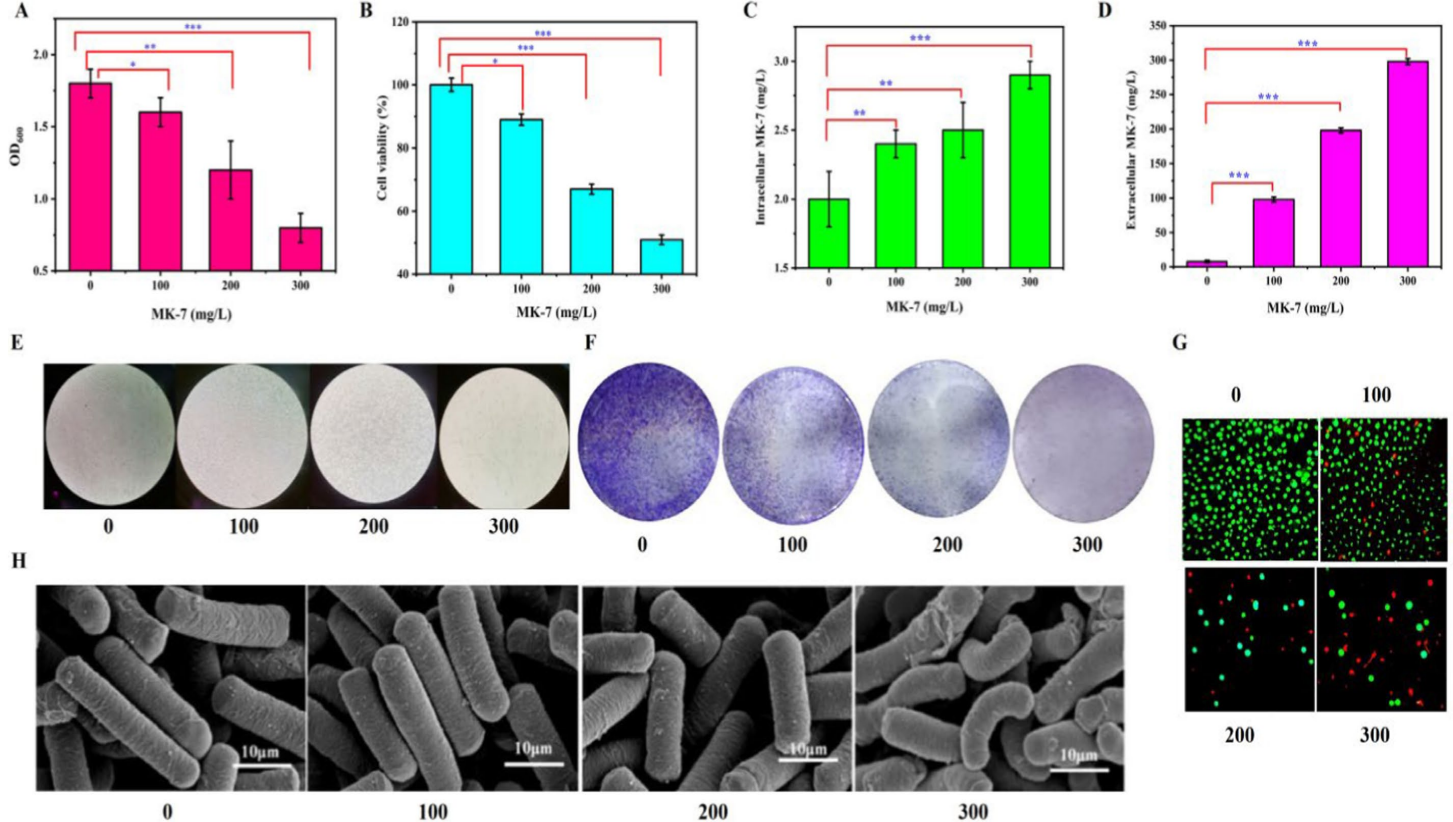
Effect of MK-7 on *Bacillus subtilis* natto. **(A)** Effect of MK-7 on *B. subtilis* natto biomass in 48 h of fermentation. **(B)** Effect of MK-7 on cell viability in 84 h of fermentation. **(C)** Effect of MK-7 on intracellular MK-7 production in 84 h of fermentation. **(D)** Effect of MK-7 on extracellular MK-7 production in 84 h of fermentation. Values and error bars represent the mean values and standard deviations of biological triplicates. **(E,F)** Ultrastructural changes to cell morphology under a regular optical microscope without **(E)** and with **(F)** crystalline violet dyeing in cells cultivated with 0, 100, 200, and 300 mg/L MK-7 in 84 h of fermentation. **(G)** Fluorescence micrographs of different concentrations of MK-7 on *B. subtilis* natto (green for living cells, red for apoptotic cells) in 84 h of fermentation. **(H)** Scanning electron microscopic images of *B. subtilis* cultivated with 0, 100, 200, and 300 mg/L MK-7 in 48 h of fermentation.

Previous studies proved that the protein complex released MK-7 in the cell membrane of *B. subtilis* natto to the external environment (Chatake et al., 2018). To detect whether an MK-7-producing strain has a good product tolerance, 0, 100, 200, and 300 mg/L MK-7 were added to the medium at the beginning of fermentation, and the cell growth and morphology of *B. subtilis* natto BN-P15-11-1 were investigated. As shown in Figures 1A,E,F, with the increase of MK-7 concentration in the medium, the OD of *B. subtilis* natto at 48 h showed a decreasing trend. The addition of 100, 200, and 300 mg/L MK-7 to the culture broth led to a 12, 34, and 56% decrease in optical density at 600 nm (OD_600_) compared to the control, indicating that the addition of extracellular MK-7 had a certain inhibitory effect on the growth of *B. subtilis* natto. The intracellular and extracellular MK-7 showed an increasing trend (Figures 1C,D). However, the increase of intracellular and extracellular MK-7 content produced by *B. subtilis* natto was not significant because extracellular MK-7 included MK-7 added in the medium.

Representative phase contrast images of cells cultivated with 0, 100, 200, and 300 mg/L MK-7 were applied to measure the effect of the target metabolite on cell viability (Figure 1G). When MK-7 was not added to the medium, the cells exhibited abundant green fluorescence with no red fluorescence. After the addition of 100, 200, and 300 mg/L MK-7 to *B. subtilis* natto, the number of cells with green fluorescence reduced significantly, while the number of cells with red fluorescence increased. Furthermore, the total number of cells in the field of view was reduced as the concentration of MK-7 increased. The addition of 100, 200, and 300 mg/L MK-7 to the medium led to a 21, 33, and 49% decrease in cell viability compared to the control (Figure 1B), indicating that higher concentrations of MK-7 induced apoptosis in *B. subtilis* natto.

The control cells, which were not cultivated with MK-7, appeared smooth, rod-shaped, and exhibited intact surface morphology. More damaged cells with broken cell walls were observed when *B. subtilis* natto was cultivated with 300 mg/L MK-7 when detected by scanning electron microscopy (Figure 1H), indicating that MK-7 could induce cellular shortening and breakage in *B. subtilis* natto.

These results demonstrated that the addition of MK-7 caused damage to cell viability in *B. subtilis* natto. Because this fat-soluble vitamin is synthesized on the cell membrane, extracellular secretion is a crucial approach for its large-scale synthesis due to the intracellular space and feedback inhibition (Wu et al., 2021). However, from the above experimental results, it can be seen that extracellular secretion exceeding 100 mg/L will negatively impact cell replication and temporal lifespan. Therefore, it is necessary to adjust the lifespan of *B. subtilis* natto to further improve the synthetic ability of MK-7.

### 3.2 Combination of SPH and SP80 could prolong the lifespan and decrease the death rate of *Bacillus subtilis* natto

**Figure 2:**
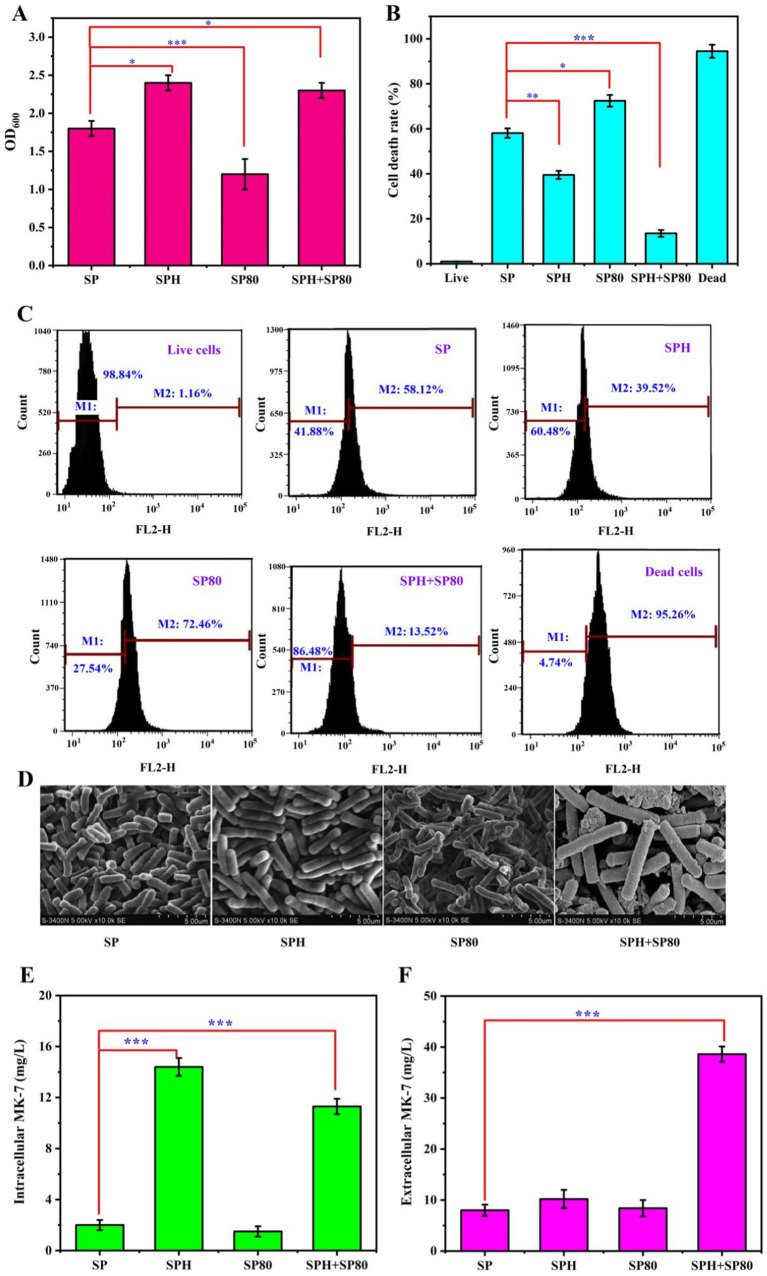
Effect of SP, SPH, SP80, and SPH+SP80 on *B. subtilis* natto. **(A)** Effect of SP, SPH, SP80, and SPH+SP80 on *B. subtilis* natto biomass in 48 h of fermentation. **(B)** Effect of SP, SPH, SP80, and SPH+SP80 on mortality rate in 84 h of fermentation. Live: negative control, which means cells were cultivated in basal fermentation medium and were not stained by PI; dead: positive control, which means cells cultivated in basal fermentation medium, destroyed by ultrasonic waves at a frequency of 20 kHz for 5 min, and stained by PI. **(C)** Survival of *B. subtilis* natto cultivated with SP, SPH, SP80, and SPH+SP80 in 84 h of fermentation, the number of apoptotic cells detected by PI-stained flow cytometry. PI is impermeable to live cells and is commonly used to detect dead cells in a population; relative cell viability is determined based on the fluorescence intensity of PI. **(D)** Scanning electron microscopic images of *B. subtilis* natto cultivated with SP, SPH, SP80, and SPH+SP80 in 48 h of fermentation. **(E)** Effect of SP, SPH, SP80, and SPH+SP80 on intracellular MK-7 production in 84 h of fermentation. **(F)** Effect of SP, SPH, SP80, and SPH+SP80 on extracellular MK-7 production in 84 h of fermentation. Values and error bars represent the mean values and standard deviations of biological triplicates.

SP was chosen as the control to evaluate the impact of the SPH-based medium for MK-7 production by *B. subtilis* natto in submerged fermentation because it was the most commonly used nitrogen source for MK-7 production (Berenjian et al., 2011). Previous studies also found that the addition of SP could adjust the cell cycle (Yi et al., 2024), and it was speculated that SPH, as a hydrolysate of SP, also played a very important role in prolonging the cell cycle. We added SP and SPH separately to the culture medium to verify this speculation and examined their effects on the RLS and CLS. It was found that the optical density at 600 nm (OD_600_) increased by 33% in the SPH group compared to the SP group (Figure 2A), indicating that the addition of SPH was more effective in increasing bacterial biomass during the stable growth period. The death rate was 39.5% when SPH was added, which was 58.1% when SP was added after 84 h of fermentation (Figures 2B,C), suggesting the bacterial mortality rate also decreased when SPH was added. Moreover, the length of individual bacterial cells significantly increased after the addition of SPH through scanning electron microscopy observation (Figure 2D). The results above suggested that SPH prolonged the RLS of cells. By examining the changes in intracellular and extracellular MK-7 content after SPH addition, it was found that the intracellular MK-7 content increased 7.2-fold, while the extracellular content just increased 1.3-fold compared to the control (Figures 2E,F). The reason may be that the significant accumulation of MK-7 within the cell inhibited bacterial metabolism (Cui et al., 2020). Previous studies found that surfactants promoted the efflux of intracellular MK-7, ultimately leading to its extensive synthesis (Liu et al., 2014). Therefore, we investigated the effects of various surfactants on the synthesis of MK-7 and, ultimately, SP80, which could be widely used in the food industry and had the greatest promoting effect on MK-7 (see Supplementary Figure S1).

By comparing the effects of SP80 and SPH+SP80 on biomass, survival rate, cell morphology, and MK-7 synthesis, it was found that the OD value of bacterial cells decreased by 34%, and bacterial length had no significant change compared to SP+SP80 (Figures 2A,D). These results indicated that SP80 itself was not conducive to bacterial RLS. However, in the SPH+SP80 group, the optical density at 600 nm (OD_600_) increased by 28% in 48 h of fermentation, and bacterial mortality decreased to 13.5% after 84 h of fermentation. Notably, the length of the *B. subtilis* natto increased significantly compared to the control (Figure 2D).

Intracellular MK-7, especially extracellular MK-7, had an obvious increase, which was 5.6-fold and 7.2-fold compared to the control (Figures 2E,F). The result was consistent with our previous study, which found that the addition of surfactant led to MK secretion into the extracellular space in *Escherichia* sp. (Liu et al., 2014). It was mentioned that the extracellular productivity was 0.46 mg/L·h^−1^ in this study, which was much higher than 0.33 mg/L·h^−1^ in that study. The reason may be that different surfactants had varying secretion abilities toward different bacteria. The total of MK-7 reached 52.9 mg/L, the highest among all studies except those involving the engineered *B. subtilis* 168 strain (Table 1). Due to the frequent loss of plasmids during the passage of genetically engineered strains in industrial production, non-genetically engineered strains were more favored. It was worth mentioning that the productivity of MK-7 reached 0.629 mg/L·h^−1^ when SPH and SP80 were added to the medium, which was the highest compared with other strategies to regulate MK-7 synthesis (Table 1). In other words, the production rate obtained under this strategy was 8.6% higher than that of the *B. subtilis* 168 engineering strain (Yang et al., 2019), indicating an increase in the MK-7 content obtained in a shorter period. Taken together, SPH+SP80 could greatly prolong *B. subtilis* natto lifespan and increase extracellular and total MK-7 content. Thus, clarifying the underlying mechanisms between nutrients and lifespan was important to achieve more stable and efficient MK-7 production in *B. subtilis* natto.

### 3.3 SPH+SP80 was more conducive for nutrient absorption in the culture medium and prolonged the *Bacillus subtilis* natto lifespan

The free amino acid composition, hydrolysis degree, surface tension, Mw distribution, and membrane integrity of SP, SP80, SPH, and SPH+SP80 were measured to analyze the mechanism for SPH+SP80 promoting the cell lifespan. Table 2 showed that the level of total amino acids in SP (265.52 g/kg) was significantly higher than that in SPH (65.89 g/kg). Especially, Phe, Arg, and Glu content, which were 23.47 ± 0.46 g/kg, 37.70 ± 4.50 g/kg, and 30.25 ± 1.17 g/kg in the SP group, could not be tested in the SPH group. As for Phe, it involved the negative feedback in MK-7 synthesis by inhibiting the.

**Table 2:** Free amino acid compositions of SP and SPH.

Amino acid	SP (g/kg)	SPH (g/kg)
Asp	9.87 ± 0.34	0.93 ± 0.09
Thr	10.46 ± 0.50	0.60 ± 0.06
Ser	11.23 ± 0.15	1.00 ± 0.09
Asn	/	1.06 ± 0.17
Glu	30.25 ± 1.17	/
Gly	10.38 ± 0.38	1.55 ± 0.12
Ala	17.84 ± 0.62	3.02 ± 0.33
Val	18.94 ± 1.49	3.76 ± 0.09
Cys	5.67 ± 0.32	4.53 ± 0.50
Met	6.06 ± 1.14	4.50 ± 0.14
Ile	15.15 ± 1.83	7.07 ± 0.23
Leu	25.30 ± 1.84	24.42 ± 0.86
Tyr	17.66 ± 1.09	4.96 ± 0.27
Phe	23.47 ± 0.46	/
His	6.03 ± 0.26	3.76 ± 0.31
Lys	15.29 ± 0.89	2.31 ± 0.23
Arg	37.70 ± 4.50	/
Pro	4.22 ± 0.64	2.42 ± 1.33
Total	265.52	65.89

**Figure 3:**
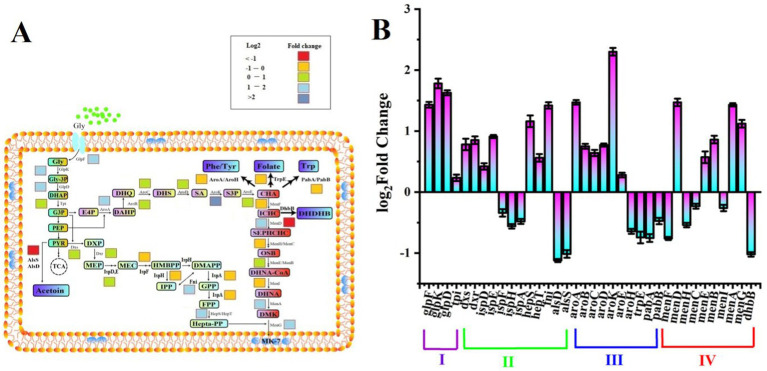
Changes in the gene expression level related to MK-7 biosynthesis between SPH+SP80 and SP groups. Biosynthetic pathway of MK-7 in *Bacillus subtilis* natto **(A)**. The level of gene expression related to MK-7 biosynthesis of SPH+SP80 was higher than that of the SP group **(B)**.

3-deoxy-arabino-heptulonate 7-phosphate synthase (AroA) is responsible for the MK-7 precursor, naphthoquinone ring, synthesis (Yang et al., 2019; Figure 3A). The concentrations of both Arg and Glu could influence global regulators between carbon and nitrogen metabolism in *Bacillus subtilis* (Sonenshein, 2007), further affecting the synthesis of metabolites, including the MK-7. In addition, Arg also showed negative effects on MK-7 production. This corresponded to the results of Arg showing a potential antimicrobial role in the dissociation of the gingivalis biofilm (Li et al., 2020).

**Figure 4:**
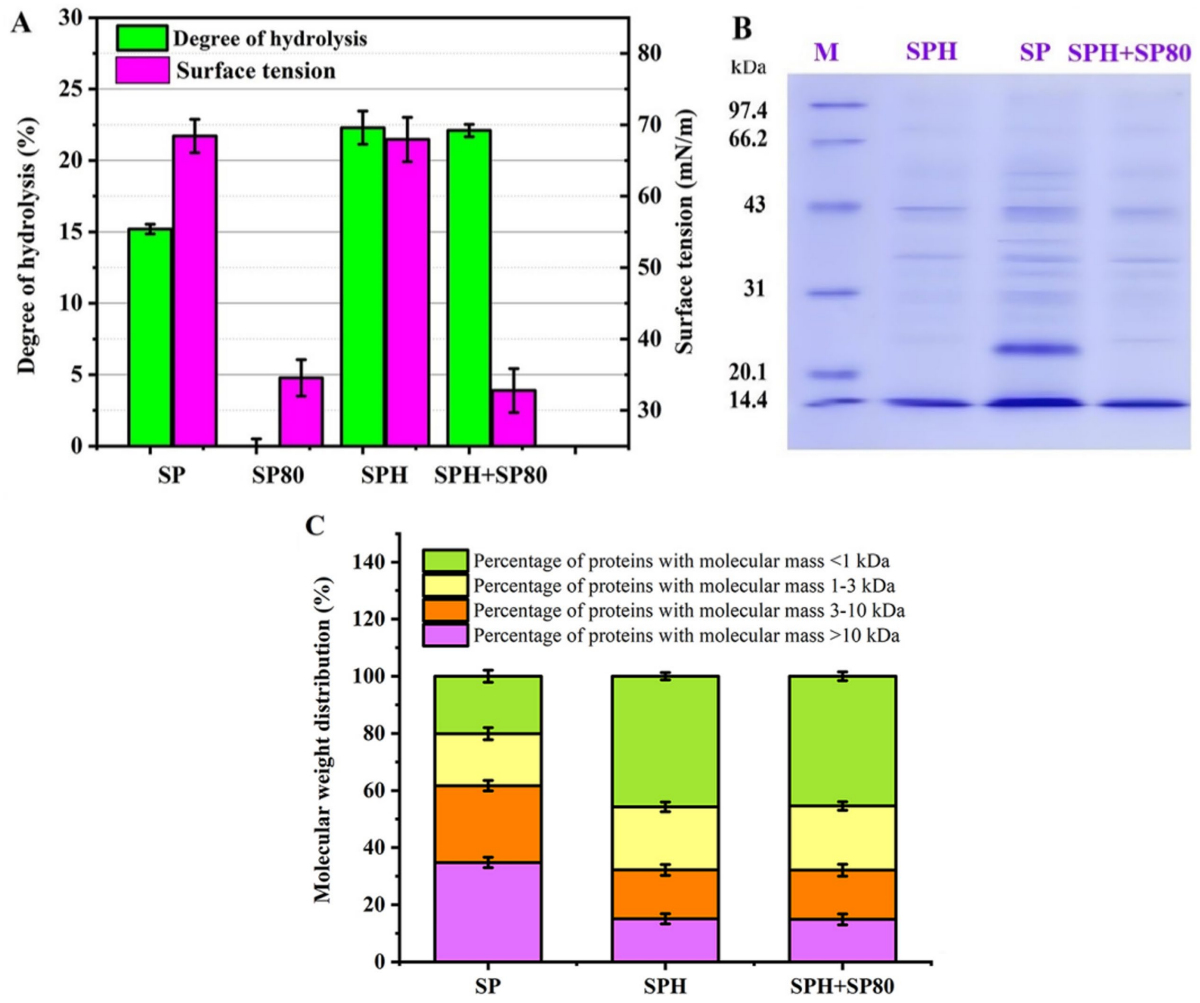
The degree of hydrolysis, surface tension, Mw distribution, and membrane permeability of SP, SPH, SP80, and SPH+SP80. **(A)** The degree of hydrolysis and surface tension for SP, SPH, SP80, and SPH+SP80. The SP, SPH, SP80, or SPH+SP80, and distilled water with a mass ratio of 1:15 were pretreated at 80°C for 10 min, and then protamex1.6 (1.5%, w/w) was added for 24 h of hydrolysis at pH 7.0, 50°C. The hydrolysates were heated to 100°C for 10 min to inactivate the enzyme and then centrifuged at 8000 *g* and 4°C for 10 min. The supernatant was obtained and tested. **(B,C)** The Mw distribution of SP, SPH, SP80, and SPH+SP80. SP, cells cultivated in basal fermentation medium supplemented with 30 g/L SP; SPH, cells cultivated in basal fermentation medium supplemented with 30 g/L SPH; SP80, cells cultivated in basal fermentation medium supplemented with 3.0 g/L SP80; SPH+SP80, cells cultivated in basal fermentation medium supplemented with 30 g/L SPH and 3.0 g/L SP80.

The hydrolysis degrees of SP, SP80, SPH, and SPH+SP80 were 15.2, 0.0, 22.3, and 22.1%, respectively (Figure 4A). More small peptides or amino acids produced by hydrolysis were conducive to fermentation (Yang et al., 2018). The surface tension of SP80 and SPH+SP80 medium decreased noticeably (34.56 mN/m, 32.78 mN/m) compared to SP and SPH medium (68.43 mN/m, 67.94 mN/m). Reduced surface tension was more conducive for nutrients to enter cells.

The Mw of SP, SP80, SPH, and SPH+SP80 groups was measured by SDS-PAGE and HPLC. More bands were visible in the SDS-PAGE gel from the SP group than from the other three groups (Figure 4B), meaning SP was composed of more high-Mw proteins (Mw > 10 kDa), which was further confirmed in Figure 4C (a 34.8% proportion of higher Mw proteins). As for SPH and SPH+SP80, the proportion of small peptides (Mw < 1 kDa) in SPH (45.7%) and SPH+SP80 (45.4%) were prominently higher than those in SP (20.1%) in the medium (Figure 4C). Because the small peptides (Mw < 1 kDa) could be directly transported into cells through a specific peptide transport system without competing with amino acids, promoting growth and stress resistance to the external environment (Jin et al., 2022), it was more efficient for cell growth and prolonging cell lifespan.

Taken together, SPH+SP80 performed better on the cell lifespan since SPH produced 2.26-fold small peptides (Mw < 1 kDa), which could be directly transported into cells through a specific peptide transport system. SP80 addition reduced surface tension and was more conducive to nutrients entering cells. Furthermore, the content of adverse amino acid components in SPH, such as Phe, Arg, and Glu, for the synthesis of MK-7 was reduced (Table 2). Further investigation was needed to elucidate the underlying mechanisms behind this phenomenon.

### 3.4 SPH+SP80 regulated the lifespan of *Bacillus subtilis* natto

**Figure 5:**
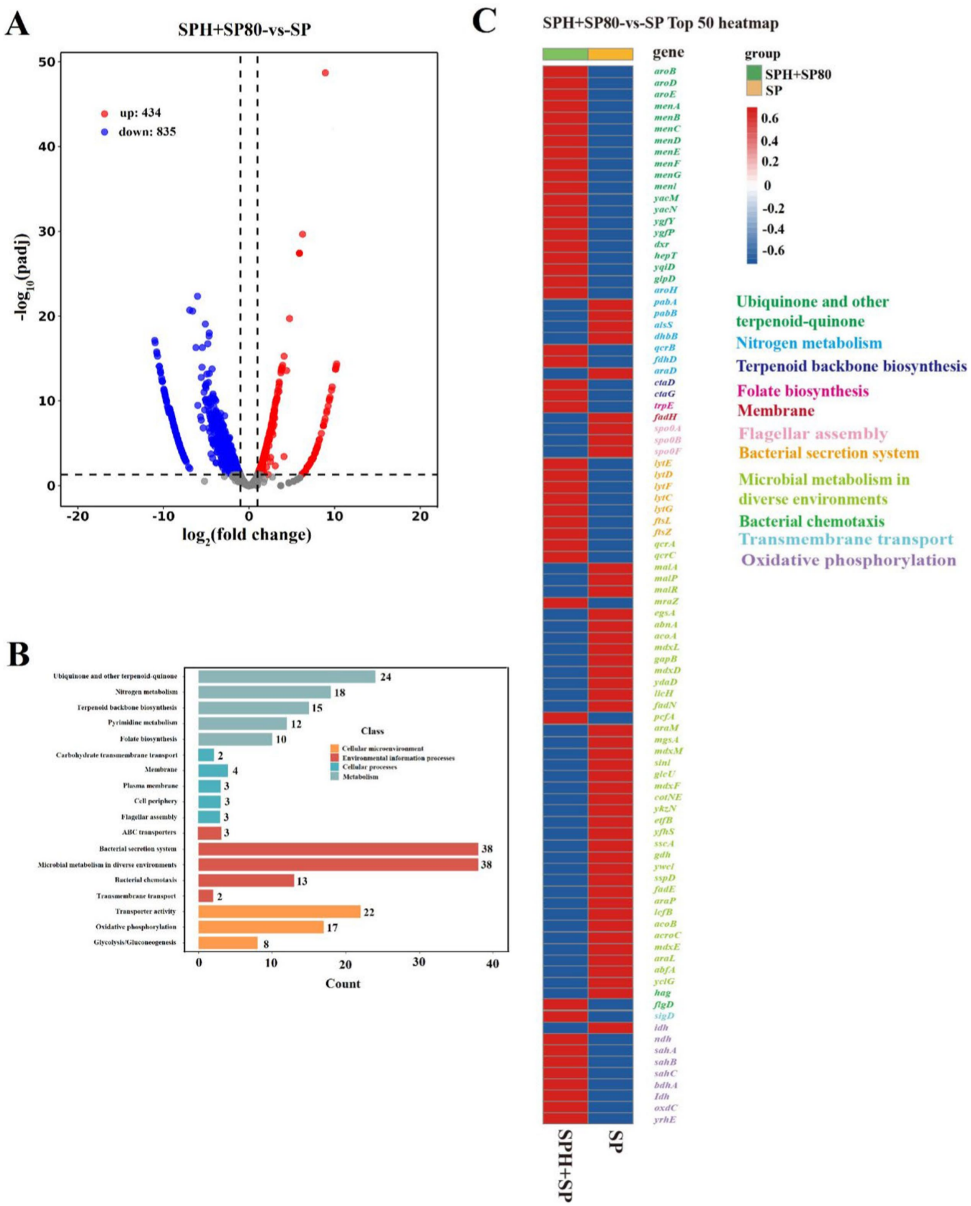
Summary of draft reads of samples by Illumina deep sequencing. **(A)** Visualization of differential genes between SP and SPH+SP80 samples by volcano plots. **(B)** Kyoto Encyclopedia of Genes and Genomes (KEGG) pathway analysis of functions of differential genes. **(C)** Top 50 of upregulated and downregulated genes of SP and SPH+SP80 samples. All experiments were independently conducted at least three times, and the results were expressed as mean ± standard deviation (SD).

To explore why SPH+SP80 regulated lifespan and MK-7 production in *B. subtilis* natto, the gene expression levels in related metabolic pathways affected by SPH and SP80 were detected by the Illumina RNAseq. The results showed significant changes in expression levels in 1269 identical genes (Figure 5A). The functional differences were mainly in “metabolism,” “cellular processes,” “cellular microenvironment,” and “environmental information processed” (Figure 5B). The top 50 genes with significant changes that belonged to these pathways included growth lifespan, ubiquinone and another terpenoid-quinone biosynthesis, oxidative phosphorylation, bacterial secretion system, microbial metabolism in diverse environments, transporter activity, and nitrogen metabolism (Figures 5B,C).

**Figure 6:**
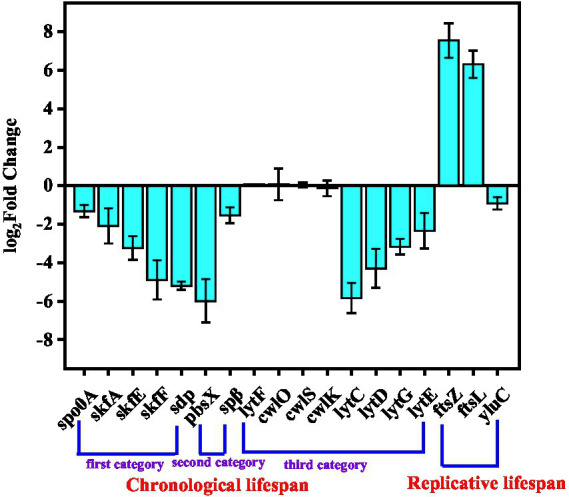
Expression level of genes related to the lifespan of cells between the SP and SPH+SP80 groups. All experiments were independently carried out at least three times, and the results were expressed as mean ± standard deviation (SD).

The different expressions of lifespan-related genes were studied to explore how SPH+SP80 regulated external morphology in *B. subtilis* natto. The first category of CLS-related genes included those involved in cannibalism (i.e., *spo0A, skfA, skfE, skfF,* and *sdp*). When spo0A was not expressed, the cells would be killed, and their contents would provide nutrients that feed their siblings and delay the sporulation of the entire bacterial population (Wang et al., 2014). The *skf* and *sdp* operons were regulated by spo0A. *skfA*, the first gene of the *skf* operon, encodes a small peptide that kills siblings. The product encoded by *skfE* and *skfF* was used to export this peptide. The *sdp* operon, which produced a signaling protein, was used to enhance the killing effect. It could be seen from Figure 6 that the expression levels of growth-related autolysis genes *spo0A, skfA, skfE, skfF,* and *sdp* were downregulated 1.31-, 2.08-, 3.23-, 4.89-, and 5.19-fold, respectively, indicating that SPH+SP80 decreased cannibalism and thus prolonged CLS.

The second category of CLS-related genes included prophages that could induce cell lysis and thus influence the lifespan of *B. subtilis* natto. Although the *B. subtilis* genome harbored at least ten putative prophages, only *pbsx* and *spβ* were lysogenic (Kunst et al., 2007). Generally, the prophage PBSX was induced to express only when treated with factors that caused survival-oriented behavior responses. *Spβ* was difficult to induce the gene expression in wild-type *B. subtilis* natto because the *c* gene encoded a strong repressor of *spβ* induction. It could be seen from Figure 6 that the expression levels of *pbsx* and *spβ* were downregulated 5.96- and 1.53-fold, respectively. Thus, SPH+SP80 eliminated the survival pressure of bacterial cells and then decreased *pbsx* expression.

The third category of CLS-related genes encoded peptidoglycan hydrolases that could also mediate cell lysis. Peptidoglycan hydrolases were encoded by the vegetative cell wall hydrolases *lytC*, *lytD*, *lytE*, *lytF*, *lytG*, *cwlO*, *cwlS*, and *cwlK*. It can be seen from Figure 6 that the expression levels of *lytF*, *cwlO*, *cwlS,* and *cwlK* had no obvious change. However, the expression levels of *lytC*, *lytD*, *lytG*, and *lytE* were downregulated 5.84-, 4.29-, 3.31-, and 2.51-fold, respectively. The reason may be that *lytC, lytD,* and *lytG* possess amidase or N-acetylglucosaminidase activities, respectively (Horsburgh et al., 2003). Meanwhile, *lytE* encoded an endopeptidase, which degrades isolated cell wall preparations.

The cell division proteins, FtsZ and FtsL, were essential cytokines associated with Z-loop maturation, facilitated the division process, and finally determined the RLS of bacteria. The zinc metalloprotease YluC was required to turn over genes *mraZ* and *ftsL* (Hashimoto et al., 2012). In our study, the expression levels of *ftsZ* and *ftsL* were upregulated by 7.52-, 6.31-fold, and the expression level of *yluC* was downregulated by 0.91-fold (Figure 6). Additionally, previous studies discovered that deletion of ftsL resulted in the decondensation of the FtsZ loop (Z-loop), preventing cells from producing more daughters (Kodama et al., 2007), and deletion of *yluC* prolonged the length of *E. coli* by approximately 33% compared to the WT (Bisicchia et al., 2007), which also verified the important role of FtsZ, FtsL, and YluC in regulating RLS of *Bacillus subtilis* natto.

Taken together, the expression level of genes that encode prophages, peptidoglycan hydrolases, the bacterial dcw cluster, and those playing roles in cannibalism during sporulation changed noticeably in the SPH+SP80 group. It not only promoted rapid cell growth, accelerated cell division, and partially delayed cell senescence of *B. subtilis* natto but also altered cellular morphology.

### 3.5 SPH+SP80 regulated the MEC, membrane potential, and electron generation system of *Bacillus subtilis* natto

The MEC was a physiological parameter that reflected the energy required to maintain intracellular homeostasis. In cells, a high MEC testified to a low energy utilization efficiency. The MEC of four strains selected from the SP, SPH, SP80, and SPH+SP80 groups was tested, and strains in the SPH+SP80 group showed the lowest MEC (0.33 mmol·g (cdw)^−1^ h^−1^) compared to the strain of the SP group, while the SP group strain had the highest MEC of 0.39 mmol·g (cdw)^−1^ h^−1^ (Table 3). Therefore, the strains of SPH+SP80 had a higher energy utilization efficiency due to their low maintenance energy metabolism. Additionally, the OD_600_ of SP, SPH, SP80, and SPH+SP80 groups also changed, with strains in the SPH+SP80 group exhibiting a rise in OD_600_ by 54.1%.

**Table 3:** Comparison of growth-related data of strains from SP, SPH, SP80, SPH+SP80 group under aerobic conditions.

Growth-related data of strains	SP	SPH	SP80	SPH+SP80
Maintenance coefficient mmol·g (cdw)^−1^ h^−1^	0.39 ± 0.03	0.35 ± 0.02	0.36 ± 0.01	0.33 ± 0.02
CDW (g/L)	1.57 ± 0.01	1.76 ± 0.03	1.28 ± 0.02	2.42 ± 0.02
Glucose consumption (g/L)	6.58 ± 0.11	6.12 ± 0.15	6.19 ± 0.12	6.02 ± 0.12
Biomass yield (g·g^−1^ glucose)	0.24 ± 0.03	0.29 ± 0.02	0.21 ± 0.04	0.40 ± 0.04
MFI	42.21 ± 1.03	84.32 ± 1.41	35.15 ± 1.16	88.23 ± 1.84

**Figure 7:**
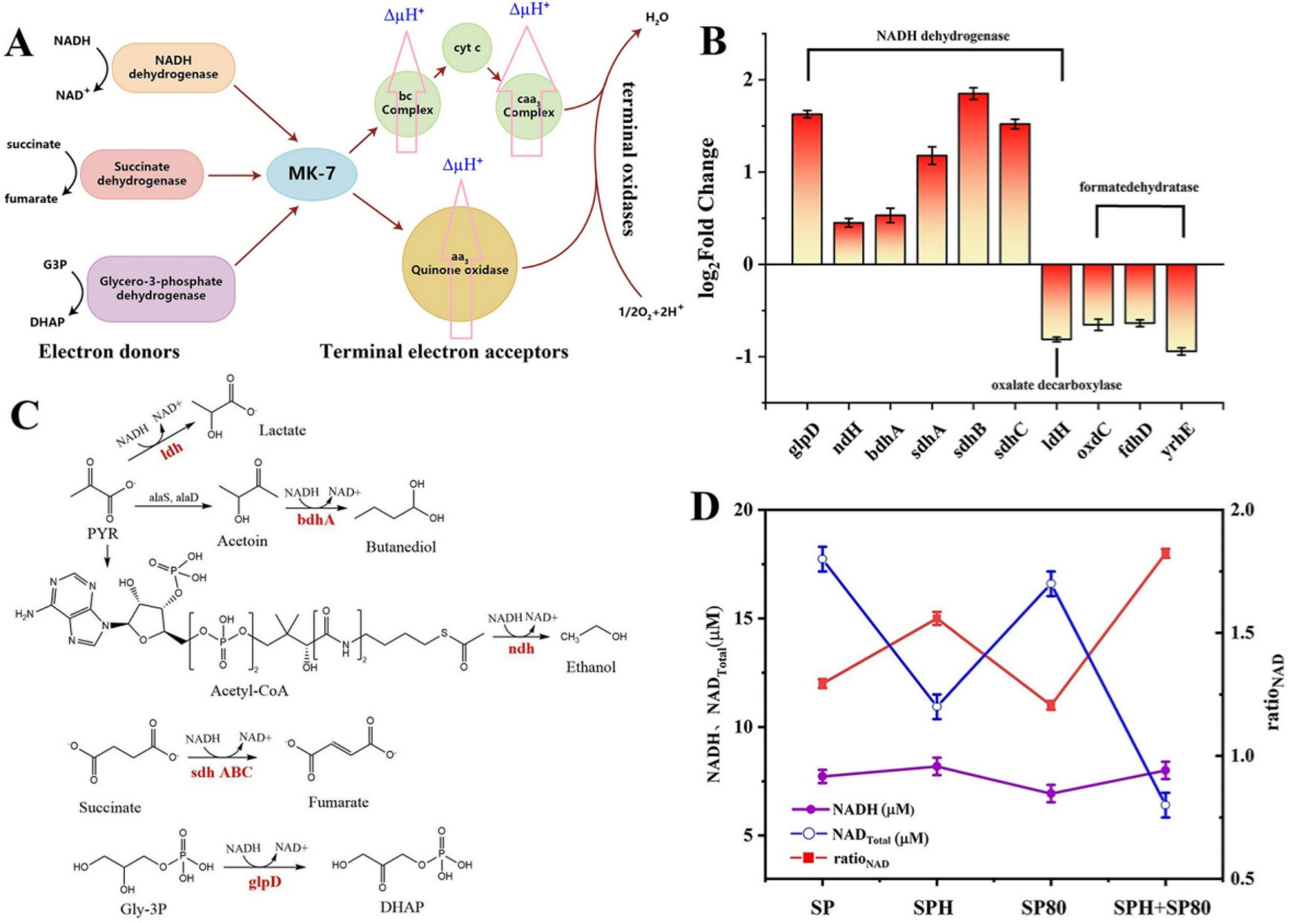
Schematic diagram of the electron transfer chain in *B. subtilis* natto. Electrons are extracted under the action of NADH dehydrogenase, succinate dehydrogenase, and glycerol 3 3-phosphate dehydrogenase. MK-7 and cytochrome *c* (Cyt *c*) act as electron transport carriers, and finally, electrons are delivered to oxygen to form water **(A)**. Changes in the membrane potential were evaluated by total NAD content, NADH content yield, and NADH/NAD^+^ ratio in SP, SPH, SP80, and SPH+SP80 groups **(B)**. Catalytic reaction of NADH reduction in cells, including l-lactate dehydrogenase (*ldH*), (R, R)-butanediol dehydrogenase (*bdhA*), NADH dehydrogenase (*ndH*), aerobic glycerol-3-phosphate dehydrogenase (*glpD*), and succinate dehydrogenase flavoprotein (*sdhABC*) **(C)**. The expression level of NADH dehydrogenase of SPH+SP80 is compared with the SP group; positive numbers mean upregulation, and negative numbers mean downregulation **(D)**. All experiments were independently carried out at least three times, and the results were expressed as mean±standard deviation (SD). G3P, glyceraldehyde-3-phosphate. DHAP, dihydroxyacetonephosphate.

MK-7 and cytochrome *c* are important components of the cell membrane and play key roles in electron transfer (Figure 7A). To investigate the effects of SP, SPH, SP80, and SPH+SP80 groups on electron transfer in the cell membrane, the membrane potential of the strain cultured for 6 days was detected. The mean fluorescence intensity (MFI) of the SPH+SP80 group (88.23 ± 1.84) increased by 2.09 times compared to the SP group (42.21 ± 1.03) (Table 3), indicating the SPH+SP80 group had electrical hyperpolarization. To explore which process causes electrical hyperpolarization, the expression level of a gene involved in encoding oxalate-decarboxylase (OxdC, catalyzing the conversion of oxalate to form electrons) was detected. Unexpectedly, the expression level of *oxdC* in the SPH+SP80 group was reduced by 65% compared to the SP group. Furthermore, the expression levels of *yrhE* and *fdhD*, which encode formate dehydratase (oxidizing formate to form electrons), were downregulated by 64 and 94% (Figure 7B), indicating that electrical hyperpolarization of the SPH+SP80 group was not due to these two processes. Therefore, we explored the other processes that could donate large amounts of electrons.

The expression levels of NADH dehydrogenases that catalyze NADH to form electrons were detected. The process of these reactions was presented in Figure 7C. Most NADH dehydrogenases were upregulated, especially *glpD*, *sdhA*, *sdhB*, and *sdhC*, which had a growth rate of 1.62-, 1.17-, 1.85-, and 1.52-fold, respectively (Figure 7B). The level of NADH, NAD^+^ total (NAD^+^ and NADH), and the ratio of NADH/NAD^+^ were also detected. The level of NAD^+^ total and NADH increased by 3.6 and 50% in the SPH+SP80 group compared to the SP group, respectively. However, the ratio of NADH/NAD^+^ reduced to 44% (Equations 1–3) (Figure 7D). The ratio of NADH/NAD^+^ has been proven to be inversely proportional to MK-7 content in *B. subtilis* (Wang et al., 2019).

### 3.6 SPH+SP80 affected the pathway related to the biosynthesis of MK-7

Four modules named glycerol metabolism pathway (Module I), methylerythritol phosphate (MEP) pathway (Module II), shikimate (SA) pathway (Module III), and MK-7 pathway (Module IV) were involved in the biosynthesis pathway of MK-7 in *B. subtilis* natto (Figure 3A; Yang et al., 2019). The different expression changes of genes involved in four modules were detected (Supplementary Table S1). In Module I, three enzymes, such as glycerol kinase (GlpK), Gly-3P dehydrogenase (GlpD), and glycerol facilitator (GlpF), played crucial roles in the SPH+SP80 group because the expression of *glpK, glpD*, and *glpF* was upregulated 1.78-, 1.62-, and 1.43-fold, respectively.

The change of most of the enzymes in Module II was not significant in the SPH+SP80 group (Figures 3A,B). Except for membrane proteins like Fni and HepS, which catalyzed a reversible reaction of isopentenyl diphosphate (IPP) to form dimethylallyl diphosphate (DMAPP), the reaction of farnesyl diphosphate (FPP) to heptaprenyl diphosphate (HDP) was upregulated 1.42-fold, and 1.16-fold, respectively.

As for Module III, the expression of *aroA*, *aroB*, *aroC*, *aroD*, *aroK*, and *aroE* was upregulated 1.47-, 0.75-, 0.64-, 0.77-, 2.30-, and 0.28-fold, respectively (Figure 3B), which verified a previous study showing that AroA and AroK played a crucial role in the SA pathway (Cui et al., 2019). The genes encoding PabA, PabB, AroH, and TrpE, which were used to synthesize aromatic amino acids such as tryptophan (Trp), tyrosine (Tyr), and phenylalanine (Phe) from chorismate, were downregulated by 36, 26, 25, and 53%, respectively.

Nine enzymes (MenA, B, C, D, E, F, G, H, and I) were involved in the synthesis of 1,4-dihydroxy-2-naphthoyl-CoA (DHNA-CoA) in Module IV. Interestingly, only genes encoding membrane proteins (*menA*, *menB*, *menD*, *menE*, and *menG*) were upregulated, especially *menA*, *menG,* and *menD,* whose expression levels were highly regulated by 1.43-, 1.12-, and 1.47-fold, respectively, in the SPH+SP80. These results provide clues for further modification of MK-7 biosynthesis at the transcriptional level.

Taken together, an active electron transfer system stimulated the expression of related genes on the cell membrane, such as *ispE*, *hepS*, *aroA*, *aroK*, *menA*, *menD*, and *menG*, which were key enzyme-encoding genes for synthesizing MK-7, and finally promoted the synthesis of MK-7.

## 4 Conclusion

In the fermentation process, *B. subtilis* natto cultivated with different nutrients exhibited significant differences in lifespan and MK-7 production. The value of OD_600_ increased by 28%, and the mortality rate decreased to 13.5% in SPH+SP80, while the death rate was 58.1% when SP was added after 84 h of fermentation. Intracellular MK-7, especially extracellular MK-7, showed a noticeable increase of 5.6-fold and 7.2-fold compared to the control (Figures 2E,F). The total MK-7 content in SPH+SP80 reached 52.9 mg/L, which was 5.3-fold compared to that in SP. The biomass and cell size were markedly enhanced by SPH+SP80, which were associated with the enhancement of cell viability, downregulation of CLS-related genes (*spo0A, skfA, skfE, skfF, sdp, pbsx*, *spβ, lytC*, *lytD*, *lytG*, and *lytE*), and the upregulation of the RLS-related genes (*ftsZ* and *ftsL*). Low Mw (<1 kDa) components and moderate amino acids, particularly Phe, Arg, and Glu in SPH+SP80, might be the predominant factors contributing to these differences. Moreover, the prolongation of the lifespan induced the MK-7 biosynthesis by upregulating the key genes on the cell membrane, such as *ispE, hepS, aroA, aroK, menA, menD*, and *menG,* related to MK-7 synthesis, therefore enhancing MK-7 precursor, isoprene side chain supply, and MK-7 assembly efficiency in *B. subtilis* natto. Briefly, SPH+SP80 reduced surface tension, enhanced nutrient absorption, and finally extended the lifespan of *B. subtilis* natto. The prolongation of the lifespan induced the MK-7 biosynthesis by upregulating the key genes on the cell membrane. For the majority of industrial microorganisms, it was difficult to produce genetically engineered bacteria due to the lack of suitable gene editing tools.

The addition of SPH and SP80 provided a potential strategy for controlling the physiological state and metabolic synthesis capabilities of industrial strains.

## Data Availability

The original contributions presented in the study are included in the article/Supplementary material, further inquiries can be directed to the corresponding authors.

## Appendix

### Abbreviations of enzymes

Module I: GlpF, glycerol uptake facilitator; GlpK, glycerol kinase; GlpD, glycerol-3-phosphate dehydrogenase; Tpi, triosephosphate isomerase.

Module II: Dxs, 1-deoxyxylulose-5-phosphate synthase; Dxr, 1-deoxyxylulose-5-phosphate reductoisomerase; IspD, 2-C-methyl-D-erythritol 4-phosphate cytidylyltransferase; IspE, 4-(cytidine 5′ -diphospho)-2-C-methyl-D-erythritol kinase; IspF, 2-C-methyl-D-erythritol 2,4-cyclodiphosphate synthase; IspG, (E)-4-hydroxy-3-methylbut-2-enyldiphosphate synthase; IspH, 4-hydroxy-3-methylbut-2- enyl diphosphate reductase; Idi, isopentenyldiphosphate delta-isomerase; IspA, which acts as geranyl pyrophosphate synthase and farnesyl pyrophosphate synthase; HepS/HepT, heptaprenyl diphosphate synthase component I/II; AlsS, acetolactate synthase; AlsD, acetolactate decarboxylase.

Module III: AroA, 3-deoxy-7-phosphoheptulonate synthase; AroB, 3-dehydroquinate synthase; AroC, 3-dehydroquinate dehydratase; AroD, shikimate dehydrogenase; AroK, shikimate kinase; AroE, 3-phosphoshikimate-1-carboxyvinyltransferase; AroF, chorismate synthase; AroH, chorismate mutase; TrpE, anthranilate synthase; PabB/PabA, para-aminobenzoate synthase component I/II.

Module IV: MenF, isochorismate synthase; MenD, 2-succinyl-5-enolpyruvyl-6-hydroxy-3-cyclohexene-1-carboxylate synthase; MenH, 2-succinyl-6-hydroxy-2,4-cyclohexadiene-1-carboxylate synthase; MenC, o-succinylbenzoate synthase; MenE, o-succinylbenzoic acid-CoA ligase; MenB, 1,4-dihydroxy-2-naphthoyl-CoA synthase; MenI, 1,4-dihydroxy-2-naphthoyl-CoA hydrolase; MenA, 1,4-dihydroxy-2-naphthoate heptaprenyltransferase; MenG, demethylmenaquinone methyltransferase; DhbB, bifunctional isochorismate lyase/aryl carrier protein.

### Abbreviations of metabolites

Module I: Gly, glycerol; Gly-3P, glycerol-3-phosphate; DAHP, 3-deoxy-arabino-heptulonate 7-phosphate; G3P, glyceraldehyde-3-phosphate.

Module II: PEP, phosphoenolpyruvate; PYR, pyruvate; DXP, 1-deoxyxylulose-5-phosphate; MEP, methyl-eryth ritol-4-diphosphate; HMBPP, 1-hydroxy-2-methyl-2-butenyl 4-diphosphate; DMAPP, dimethylallyl diphosphate; IPP, isopentenyl diphosphate; GPP, geranyl diphosphate; FPP, farnesyl diphosphate.

Module III: E4P, erythrose 4-phosphate; CHA, chorismate; DHQ, 3-dehydroquinate; DHS, 3-dehydroshikimate; SA, shikimate; S3P, shikimate 3-phosphate; Phe, phenylalanine; Tyr, tyrosine; Trp, tryptophan.

Module IV: ICHA, isochorismate; SEPHCHC, 2-succinyl-5-enolpyruvyl-6-hydroxy-3-cyclohexene-1-carboxylate; OSB, 2-succinylbenzoate; DHNA-CoA, 1,4-dihydroxy-2-naphthoyl-CoA; DHNA, 1,4-dihydroxy-2-naphthoate; DMK, 2-demethylmenaquinone; MK-7, menaquinone-7; DHDHB, (2S,3S)-2,3-dihydro-2,3-dihydroxybenzoate.
