# Supplementary material for: Combination of SPH and SP80 prolongs the lifespan of Bacillus subtilis natto to enhance industrial menaquinone-7 biosynthesis
Source: Front Microbiol. 2025 Jun 4;16:1578160. doi: 10.3389/fmicb.2025.1578160 (PMC12174048; doi:10.3389/fmicb.2025.1578160)
Supplement: Supplementary file 1 [file Table_1.docx]

**Supplementary material**

Fig.S1. Effects of different surfactants on the production of intracellular MK-7, extracellular MK-7, and cell growth. All experiments were carried out in triplicate and data are expressed as mean values±S.D. Concentrations of surfactants (in g/L): alkyl dimethyl benzyl ammonium chloride (ADBAC) 3.0, betaine 3.0, polyoxyethylene oleyl ether (POE) 3.0, sodium dodecyl benzene sulphonate (SDBS) 3.0 and Span 80 3.0.
